# Independent genetic basis of meiotic crossover positioning and interference in domestic pigs

**DOI:** 10.1038/s41598-025-93003-7

**Published:** 2025-03-18

**Authors:** Cathrine Brekke, Arne B Gjuvsland, Peer Berg, Susan E Johnston

**Affiliations:** 1 Institute of Ecology and Evolution, School of Biological Sciences, University of Edinburgh, Charlotte Auerbach Road, Edinburgh, EH9 3FL UK; 2 Department of Animal and Aquacultural Sciences, Norwegian University of Life Sciences, Oluf Thesens vei 6, Ås, 1433 Norway; 3 Norsvin, Storhamargata 44, Hamar, 2317 Norway; 4 Geno, Storhamargata 44, Hamar, 2317 Norway

**Keywords:** Evolutionary genetics, Genetic association study, Meiosis

## Abstract

Meiotic crossover patterning shows huge variation within and between chromosomes, individuals, and species, yet the molecular and evolutionary causes and consequences of this variation remain poorly understood. A key step is to understand the genetic architecture of the crossover rate, positioning, and interference to determine if these factors are governed by common or distinct genetic processes. Here, we investigate individual variation in autosomal crossover count, crossover position (measured as both intra-chromosomal shuffling and distance to telomere), and crossover interference in a large breeding population of domestic pigs (*N* = 82,474 gametes). We show that all traits are heritable in females at the gamete (h^2^ = 0.07–0.11) and individual mean levels (h^2^ = 0.08–0.41). In females, crossover count, and interference are strongly associated with *RNF212*, but crossover positioning is associated with *SYCP2*, *MEI4*, and *PRDM9*. Our results show that crossover positioning and rate/interference are driven by distinct genetic processes in female pigs and have the capacity to evolve independently.

**Supplementary Information:**

The online version contains supplementary material available at 10.1038/s41598-025-93003-7.

## Introduction

Meiotic recombination through chromosomal crossovers is a fundamental feature of sexually reproducing species. It is required for the proper segregation of chromosomes into eggs and sperm, with most species having a minimum requirement of one crossover per chromosome pair^1^. It also facilitates adaptation by bringing together beneficial alleles at linked loci^2^, and provides a mechanism to purge deleterious alleles from genomes^3^. However, the crossover process also has risks, as it increases the rate of mutations at double strand break (DSB) repair sites^4^and can uncouple beneficial alleles at linked loci^5^. The genetic mechanisms of recombination and crossing-over are highly conserved across eukaryotes, yet there is a high diversity in their sequence and function^6,7^. Furthermore, the rate and distribution of crossovers show a huge diversity both within and between chromosomes, individuals, sexes, populations and species^8,9^. A key step in understanding this diversity is to determine its genetic architecture at the individual level (i.e., the underlying gene variants and their relative effects on crossover processes), as this can help identify the molecular mechanisms underpinning variation, its evolutionary capacity and constraints, and potential effects on downstream evolutionary processes^8,10^.

To gain a full picture of the causes and consequences of crossover variation, there are several non-independent processes that must be considered. First, we must consider ***how many crossovers occur***. Fewer crossovers may increase the risk of non-disjunction, whereas too many may lead to genome instability and increased rates of deleterious mutation^1^. The genetic basis of autosomal crossover count has been investigated in several mammal species, including humans, cattle, pigs, sheep, and deer^11–20^. A number of large effect loci have been identified, including *RNF212*, *RNF212B*, *MEI1*, *MSH4*, *PRDM9*, and *REC8*, among others, with their functions associated with meiotic processes such as crossover designation and DSB initiation and repair^6^. In particular, the locus *RNF212* and/or its paralogue *RNF212B*are consistently associated with crossover count variation in almost all of these studies, and likely exhibits a dosage dependent effect on crossover number^21^.

Second, we must consider ***where crossovers occur***. The positioning of crossovers will influence the proportion of alleles on a chromosome that will be coupled or uncoupled (or “shuffled”). For example, a crossover situated at the end of a chromosome pair will shuffle a relatively small proportion of alleles as most linked variants will remain intact, whereas a crossover situated in the centre of a chromosome pair will shuffle a relatively high proportion of alleles, as all loci on one side of the crossover are uncoupled with those on the other side^22^. This distinction is likely to have evolutionary consequences in terms of the rate of generation of novel linked allelic variation within populations^22^. Patterns of crossover positioning are likely to be affected by the physical structure of meiotic chromosomes. During meiosis, the DNA is structured into chromatin loops tethered along an axis structure, and held in close proximity by a protein structure called the synaptonemal complex (SC)^23^, and shorter axes/SCs and longer DNA loops are associated with reduced crossover rates^24^. Similarly, crossovers can vary relative to local chromatin accessibility, functional genetic elements, methylation patterns, structural variants, and proximity to centromeres and telomeres^9,25,26^. The genetic basis of fine-scale recombination landscapes in mammals has been well documented, and is most often mediated by the rapidly evolving locus *PRDM9*, whose protein binds to particular allele-specific DNA sequence motifs and promotes DSB formation in recombination “hotspots” of around 1–10kb in width^26,27^. However, much less is known about *individual*variation in broad-scale crossover positioning in mammals, although heritable variation has been identified in house sparrows and Atlantic salmon^28,29^and heritable changes in crossover landscapes have been observed under domestication in tomatoes, rye and barley^30–32^.

Third, we must consider ***how crossovers interfere***with one other. The distribution of crossovers along a chromosome is affected by the phenomenon of “crossover interference”, where a crossover forming in one position will reduce the probability that more crossovers will form nearby^33^. Several mechanisms have been proposed to explain how and why crossover interference is pervasive across eukaryotes, relating to mechanical stress on the chromosome, telomere initiation of crossovers, and aggregation of pro-crossover proteins into coarse clusters (“coarsening model”), among others^34–36^. Crossover interference is likely to be affected by chromosome length and structure, where shorter chromosomes, axes/SCs and larger DNA loops may increase the effects of interference at the base-pair scale^24,33^. Heritable variation in individual crossover interference has been identified in cattle, with some variance explained by the locus *NEK9*^37^. However, other such studies remain rare, as analyses rely on information from two or more crossovers on the same chromosome which are infrequent at the genome-wide scale, meaning that large sample sizes per individual are required to measure this metric with accuracy^10^. In *Arabidopsis thaliana*, the locus *Hei10* (in the same E3 ubiquitin ligase family and with a similar function to *RNF212*) has been associated with crossover interference through dosage along the chromosome, corresponding to the coarsening model described above^36^.

### All three of these processes interact

Crossover interference will affect how many crossovers can be placed on the chromosome; the number of crossovers will also affect levels of allelic shuffling; and crossover positioning will determine if there is enough distance for other crossovers to overcome interference. Yet, it remains unclear the degree to which these three phenomena are phenotypically and genetically correlated, and in turn, if these processes have the capacity to evolve independently. Understanding these distinctions may have importance in separating the mechanistic and evolutionary drivers of recombination e.g. through ensuring fertility through obligate crossing over, or through novel haplotype generation/haplotype conservation via crossover positioning. We must also consider that crossover processes are occurring within two distinct gametic environments: oogenesis and spermatogenesis. Differences in crossover rates and distribution between the sexes is a near universal phenomenon in eukaryotes (known as “heterochiasmy”, or “achiasmy” when recombination is absent in one sex^38,39^), and there is increasing evidence in vertebrates that the genetic architecture of crossover processes is also different between the sexes^11,16,19,28,29^. Therefore, determining phenotypic and genetic variation and correlations in these processes, in both males and females, will allow us to have a better understanding of the molecular mechanisms, evolutionary potential, and evolutionary constraints operating on these processes^10^.

Domestic pigs represent an excellent system to explore variation in crossover processes, as large litters allow the quantification of crossover counts, positioning, and interference in both males and females. Previous studies have shown that gamete crossover count in pigs is heritable, with a polygenic architecture in males and an oligogenic architecture in females associated with variants at *RNF212*, *SYCP2*, *PRDM9*, *MEI1* and *MSH4*^15,18^. However, the genetic basis of crossover positioning and interference, and their phenotypic and genetic relationship with crossover count, remains unknown. In this study, we integrate SNP data with a large breeding pedigree of Large White pigs to quantify crossover positions in gametes transmitted from parents to offspring. We quantify crossover count, crossover positioning, and crossover interference, and determine the heritability, genetic correlations, and genetic architecture of these traits within each sex. We show that crossover count and interference show a shared genetic architecture in females, mostly driven by variants at *RNF212*, whereas crossover positioning is independent of *RNF212* and instead mediated by variants at the synaptonemal complex proteins *MEI4* and *SYCP*2, and the fine-scale recombination landscape locus *PRDM9*.

## Results

### Crossover phenotype dataset

We used data from a breeding pedigree of Large White pigs genotyped at 50,705 SNP markers to characterise autosomal crossover positions in gametes transmitted from focal individuals to their offspring. SNP positions are known relative to the Sscrofa11.1 reference genome. Our dataset comprised of 41,237 gametes transmitted from 4,704 unique female FIDs, and 41,237 gametes transmitted from 271 unique male FIDs. We used the crossover positions to estimate the four crossover phenotypes across all autosomes within each gamete (detailed descriptions of the calculation of all phenotypes are provided in the methods section):

- ***Crossover Count***: the total number of autosomal crossovers within a gamete.
- ***Intra-Chromosomal Allelic Shuffling (*** $\:{\stackrel{-}{\varvec{r}}}_{\varvec{i}\varvec{n}\varvec{t}\varvec{r}\varvec{a}}$*)*: the probability that a random pair of loci on a chromosome are reshuffled during meiosis because of a recombination event (see Veller et al.^22^). This metric is partially influenced by the number of crossovers, but more so by the position of the crossover; a crossover closer to the centre shuffles more pairs of loci and gives a higher value of $\:{\stackrel{-}{r}}_{intra}$, whereas a crossover occurring towards chromosome ends will give a lower value.
- ***Distance to Telomere (Mb)***: the mean distance between the telomere and the closest crossover on the same chromosome arm within a gamete.
- ***Crossover Interference (*n*)***: the strength of crossover interference as a composite of all gametes transmitted from an FID, as defined by the shape parameter n. This was fitted with a Houseworth-Stahl interference escape model, which models both interfering (Class I) and non-interfering (Class II) crossovers^40^.

**Fig. 1:**
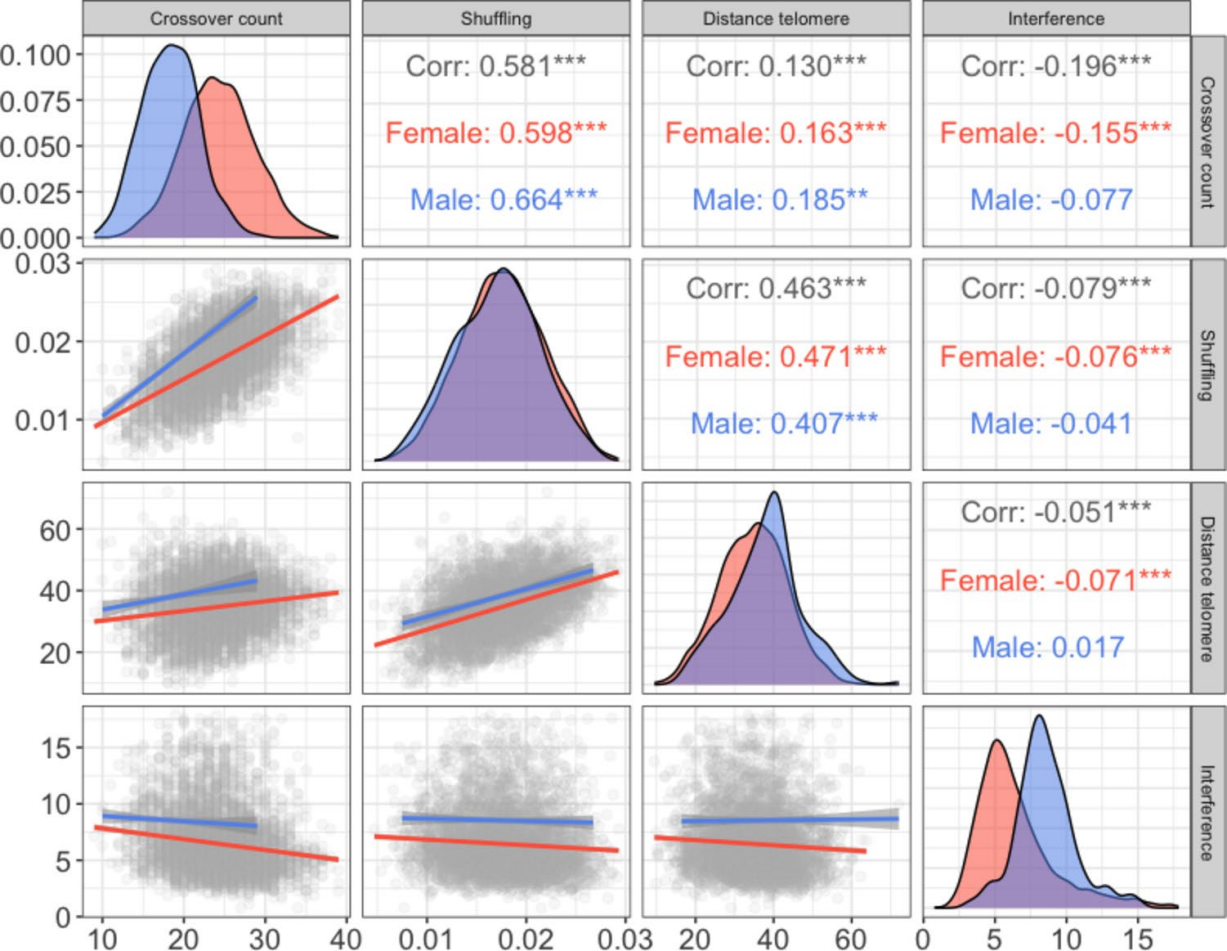
Sex-specific distributions of crossover phenotypes (diagonal panels) and their sex-averaged and sex-specific Pearson correlations (upper triangle) and linear regressions (lower triangle). Each grey point indicates an individual gamete. Male and female phenotypic correlations and distributions are indicated in blue and red, respectively. *** and ** indicate correlation significances of *P* < 0.001 and *P* < 0.01, respectively; no asterisks indicate *P* > 0.05. The shaded area around the regression lines indicates the 95% confidence interval.

All autosomal crossover phenotypes showed significant differences in their mean between female and male gametes (*P* < 0.001; Table 1; Fig. 1, Table S1). Crossover counts were higher in female gametes, but distance to telomere and crossover interference were higher in male gametes (Table 1; Fig. 1). Male mean crossover counts ranged from 14 to 26, and female mean crossover counts ranged from 12 to 43, consistent with individuals having at least one crossover per bivalent per meiosis. Both males and females showed positive crossover interference, i.e., crossovers are more distantly spaced than expected by chance; interference was stronger in males than in females (Table 1; Fig. 1). The level of interference was similar to other mammal systems, such as humans, cattle, sheep, and mouse, which range from *n* = 6.7 in female cattle to *n*= 11.65 in male mouse^33^. A small proportion of crossovers were designated as non-interfering (i.e., Class II crossovers) although this was not significantly different from zero in either sex (4.9% and 4.3% of crossovers in males and females respectively, SE = 6%).

Crossover count and intra-chromosomal shuffling were positively phenotypically correlated (Pearson’s *r* = 0.581, *P* < 0.001), as were crossover count and the distance to telomere (*r* = 0.130, *P* < 0.001), and intra-chromosomal shuffling with distance to telomere (*r* = 0.463, *P* < 0.001). Crossover interference was weakly negatively correlated with all other phenotypes in female gametes (*r* = −0.155 to −0.071; *P* < 0.001) and was not correlated with other phenotypes in male gametes (*P* > 0.05, Fig. 1).

### Heritability and genetic correlations of crossover measures

The heritability of each crossover phenotype within each sex was estimated using an animal model approach^41^. Heritabilities for crossover rate and position metrics were estimated for (A) individual gamete phenotypes, with individual FID fitted as an additional random “permanent environment effect”, and (B) mean phenotype of all gametes of an FID (or composite phenotype in the case of crossover interference). In females, all crossover phenotypes were significantly heritable (individual gamete h^2^ = 0.07–0.11, individual mean h^2^ = 0.08–0.41; Table 1). In males, all crossover phenotypes in individual gametes were significantly heritable (h^2^ = 0.03–0.06; Table 1A). However, the relatively small number of male FIDs (*N* = 271) led to higher uncertainty of heritability estimates of mean phenotypes (Table 1B). Male crossover interference was not.

**Table 1:** **Sample sizes**,** means**,** and estimated variance components for all Crossover phenotypes for males (M) and females (F).** Models were run for (A) individual gamete phenotypes and (B) the individual mean or composite phenotypes (i.e. Crossover interference) across all of their gametes. N_FIDs_ is the number of unique focal individuals, N_obs_ is the total number of observations, V_P_is the phenotypic variance, h^2^is the narrow-sense heritability, pe^2^ is the proportion of permanent environmental variance (i.e., FID identity), and r_A_ is the genetic correlations between males and females. Values in parenthesis are the standard errors. P-values for differences in female and male means are provided in table S1.

A) Individual gamete phenotypes								
Crossover Phenotype	Sex	*N*_FIDs_	*N*_obs_	Mean	V_*P*_	h^2^	pe^2^	Cross-sex *r*_A_
Crossover Count	M	271	41,237	18.20 (3.37)	11.43 (0.11)	0.058 (0.007)	0.43e^−10^ (0.42e^−12^)	0.48 (0.10)
	F	4,704	41,237	24.37 (4.58)	21.04 (0.17)	0.105 (0.009)	0.11e^−3^ (0.02)	
Intra-Chr Allelic Shuffling ($\:{\stackrel{-}{r}}_{intra}$)	M	271	41,237	0.0172 (0.004)	0.16e^−4^ (0.14e^−6^)	0.039 (0.010)	0.40e^−2^ (0.02)	0.26 (0.14)
	F	4,704	41,237	0.0175 (0.004)	0.18e^−4^ (0.14e^−6^)	0.087 (0.009)	0.02 (0.02)	
Distance to Telomere (Mb)	M	271	41,237	37.25 (9.48)	90.19 (0.75)	0.029 (0.009)	0.01 (0.01)	0.18 (0.14)
	F	4,704	41,237	34.80 (8.65)	74.90 (0.57)	0.066 (0.007)	0.01 (0.01)	
B) **Individual mean/composite phenotypes**								
Crossover Count	M	271	271	18.21 (1.23)	0.49 (0.46)	0.856 (0.142)	-	*
	F	4,704	4,704	24.31 (2.67)	8.61 (0.47)	0.412 (0.038)	-	
Intra-Chr Allelic Shuffling ($\:{\stackrel{-}{r}}_{intra}$)	M	271	271	0.0173 (0.001)	2.06e-06 (6.47e-07)	0.519 (0.164)	-	*
	F	4,704	4,704	0.0176 (0.002)	8.33e-06 (3.64e-07)	0.305 0.035	-	
Distance to Telomere (Mb)	M	271	271	37.56 (3.50)	19.13 (3.40)	0.220 (0.135)	-	*
	F	4,704	4,704	34.92 (4.79)	33.98 (1.45)	0.275 (0.035)	-	
Crossover Interference (ν)	M	271	271	8.54 (2.07)	12.76 (1.10)	0.000 (0.000)	-	*
	F	4,704	4,704	6.45 (2.77)	24.24 (0.70)	0.080 (0.022)	-	

*models did not converge for cross sex correlations on individual mean measures due to a low number of males.

**Table 2:**
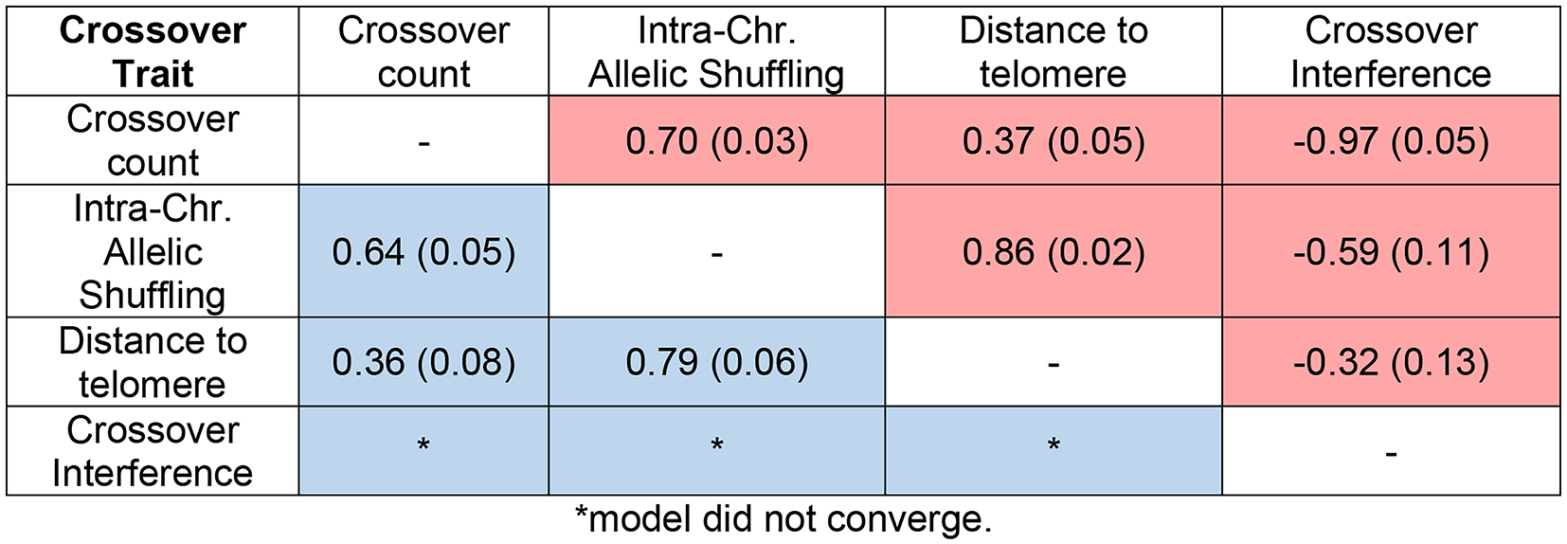
Additive genetic correlations between individual gamete crossover phenotypes within each sex. Female correlations are shown in the upper triangle and male correlations are shown in the lower triangle. Values in parentheses are the standard errors.

heritable, and while male crossover count appeared to be highly heritable (h^2^ = 0.86), there was very low phenotypic variance for this trait (V_P_ = 0.49 relative to mean = 18.21), in turn meaning that additive genetic variance was also very low.

Cross-sex genetic correlations were modelled for all individual gamete phenotypes and crossover interference; these were positive and low to moderate in magnitude (Table 1A). Intra-chromosomal allelic shuffling and distance to telomere both showed relatively low cross-sex additive genetic correlations (r_A_ = 0.26 and 0.18, respectively; Table 1A), despite no phenotypic sex-differences in mean values (Fig. 1, Table 1A), indicating the genetic architecture of these traits are largely independent between the sexes. Within each sex, crossover count, intra-chromosomal allelic shuffling and distance to telomere had significant positive additive genetic correlations (r_A_ = 0.36–0.86), whereas crossover interference in females had negative additive genetic correlations with all other crossover phenotypes (r_A_ = −0.32 to −0.97; Table 2). Crossover count and interference showed a very strong negative additive genetic correlation (r_A_ = −0.97, Table 2), indicating a strongly shared genetic architecture. We did not estimate additive genetic correlations between male crossover interference and other crossover traits due to the absence of heritable variation.

## Genome-wide association studies (GWAS)

**Fig. 2:**
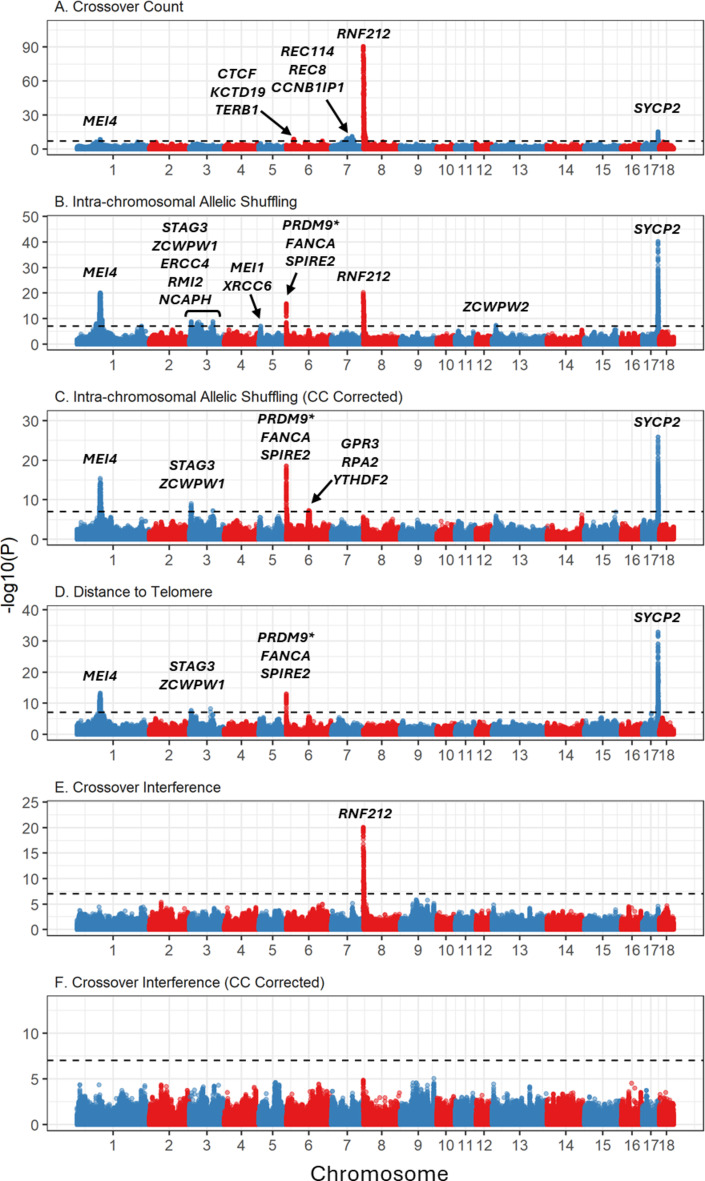
Genome-wide association plots for 6 crossover traits (Panels A-F) in females. Associations are displayed at 554,768 SNPs relative to the domestic pig genomic Sscrofa11.1. Sample sizes are provided in Table 1. CC Corrected indicates that crossover count was fitted as a fixed covariate in the model. Gene names above significant peaks indicate direct candidate loci. The dashed line indicates the genome-wide significance threshold at α = 0.05. Association statistics have been corrected with the genomic control parameter λ. Information on significant loci and candidate genes are provided in Table 2 and Tables S2-4. Plots of significant regions are provided in Figure S1. *PRMD9 is annotated as *PRDM7* on Sscrofa11.1; see Discussion.

Genome-wide association studies were carried out using a larger imputed dataset of 524,587 SNPs and were run within each sex separately on individual mean phenotypes. All crossover phenotypes had significant associations in the GWAS studies in females, whereas there were no significant associations in males (Fig. 2; Table 3). For all significant associations, we identified candidate genes in the proximity of the most highly associated SNPs based on gene ontology (GO) terms associated with meiotic processes. All significant SNP associations are provided in Table S2. Individual plots of significant regions are provided in Figure S1. All candidate gene positions are provided in Table S3, with associated GO terms provided in Table S4. In cases where a phenotype had more than one significantly associated locus, we modelled genotype-by-genotype interactions, but none were significant (Table S5). We describe results for each phenotype in detail:

### Crossover count

This trait has been investigated previously in the same dataset^15^, but was reanalysed here using the leaving-one-chromosome-out model (see methods for details), which identified new associations. A GWAS identified six regions on five chromosomes that were significantly associated with female crossover count, corresponding to ten candidate genes. The strongest association was observed at *RNF212* on chromosome 8 (Fig. 2A, Figure S3, Table 3). Significant associations were also identified at two regions in common with intra-chromosomal allelic shuffling and distance to telomere, corresponding to *MEI4* and *SYCP2* on chromosomes 1 and 17, respectively. There were three regions uniquely associated with crossover count, including a region on chromosome 6 corresponding to the candidate genes *CTCF*, *KCTD19* and *TERB1*, and a region on chromosome 7 corresponding to the candidate genes *REC114*, *REC8* and *CCNB1IP1*.

### Intra-chromosomal allelic shuffling (Crossover positioning)

GWAS identified significant associations in ten regions on seven chromosomes, corresponding to 18 potential candidate genes, including *RNF212* (Fig. 2B; Table 3). As intra-chromosomal allelic shuffling is also affected by crossover count, we ran an additional GWAS including crossover count fit as a fixed covariate in the model (Fig. 2C; Table 3). After this correction, only six regions on 4 chromosomes remained significant, and the association with *RNF212* was no longer present. The strongest association was observed at *SYCP2* on chromosome 17, with additional strong associations shown at *MEI4* on chromosome 1, and *PRDM9*, *FANCA* and *SPIRE2* on chromosome 6. Note that *PRDM9* on the pig genome is annotated as *PRDM7*; however, we hereafter refer to this locus as *PRDM9*, based on identification and discussion in previous publications, and high conservation of sequence and function between *PRDM7* and *PRDM9*^18,27,42^.

### Distance to telomere

This trait was highly correlated with intra-chromosomal shuffling in each sex (r_A_ = 0.79 and 0.86 in males and females, respectively), but less so with crossover count (r_A_ = 0.36 and 0.37 in males and females, respectively; Table 2). GWAS identified significant associations in five regions on 4 chromosomes (Fig. 2D; Table 3). Similarly to intra-chromosomal allelic shuffling, the strongest association was observed at *SYCP2* on chromosome 17, with additional strong associations shown at *MEI4* on chromosome 1, and *PRDM9*, *FANCA* and *SPIRE2* on chromosome 6.

### Crossover interference

Only one region was significantly associated with female crossover interference, corresponding to *RNF212* on chromosome 8 (Table 2; Fig. 2E). This association was no longer significant when fitting crossover count as a fixed covariate in the model (Fig. 2F); combined with a strong negative genetic correlation (r_A_ = −0.97), this indicates that *RNF212* has a strong effect on both crossover count and crossover interference in females of this population.

**Table 3:** Genomic regions associated with crossover phenotypes in females. Information is provided for the most highly associated SNP loci; full results are in table S2. MAF is the minor allele frequency. B is the effect size of the minor allele with standard error SE. P is the association significance after correction for genomic control. Candidate genes are those directly associated with meiotic processes. SNP positions are relative to the pig genome Sscrofa11.1. Full gene names, positions, and gene ontology terms are in tables S3 and S4.

Crossover Trait	Chr	Top SNP(s) Position	MAF	B	SE	*P*	Candidate Genes
Crossover Count	1	88,682,309	0.337	−0.385	6.51E-02	3.48E-09	*MEI4*
	6	28,699,461	0.194	0.450	7.55E-02	2.62E-09	*CTCF*,* KCTD19*,* TERB1*
	6	137,764,455	0.246	0.410	7.65E-02	7.91E-08	*MSH4*
	7	78,531,203	0.385	0.434	6.32E-02	6.25E-12	*REC114*,* REC8*,* CCNB1IP1*
	8	164,462 to 244,327	0.287	−1.389	6.86E-02	4.88E-91	*RNF212*
	17	59,902,573	0.459	−0.485	6.01E-02	6.97E-16	*SYCP2*
Intra-chromosomal Allelic Shuffling ($\:{\stackrel{-}{r}}_{intra}$)	1	89,299,915	0.342	−0.055	5.88E-03	8.91E-21	*MEI4*
	1	245,271,070	0.164	0.040	7.57E-03	8.88E-08	*SMC2*
	3	8,180,895	0.373	0.033	5.40E-03	1.49E-09	*STAG3*,* ZCWPW1*
	3	36,338,816	0.464	0.033	5.51E-03	2.23E-09	*ERCC4*,* RMI2*,* NCAPH*
	3	90,716,599	0.488	−0.032	5.37E-03	1.90E-09	*MSH2*,* MSH6*
	5	6,572,211	0.294	0.033	6.09E-03	7.91E-08	*MEI1*,* XRCC6*
	6	244,884	0.329	−0.047	5.69E-03	1.33E-16	PRDM9*,* FANCA*,* SPIRE2*
	8	256,579	0.294	−0.057	6.12E-03	6.49E-21	*RNF212*
	13	14,861,969	0.195	0.039	7.05E-03	4.42E-08	*ZCWPW2*
	17	59,911,489 to 59,968,884	0.462	−0.072	5.42E-03	8.59E-41	*SYCP2*
Intra-chromosomal Allelic Shuffling (CC Corrected)	1	89,299,915	0.342	−0.038	4.63E-03	4.23E-16	*MEI4*
	3	8,180,895	0.373	0.026	4.27E-03	1.01E-09	*STAG3*,* ZCWPW1*
	3	90,716,599	0.488	−0.023	4.23E-03	5.99E-08	*MSH2*, MSH6**
	6	156,711	0.360	−0.040	4.42E-03	2.78E-19	PRDM9*,* FANCA*,* SPIRE2*
	6	85,326,680	0.357	−0.024	4.41E-03	5.44E-08	*GPR3*,* RPA2*,* YTHDF2*
	17	59,911,489 to 59,968,884	0.462	−0.046	4.27E-03	1.27E-26	*SYCP2*
Distance to Telomere (Mb)	1	88,051,896	0.322	−0.860	1.14E + 05	5.89E-14	*MEI4*
	3	8,180,895	0.373	0.601	1.07E + 05	2.08E-08	*STAG3*,* ZCWPW1*
	3	81,421,111	0.246	−0.739	1.26E + 05	4.92E-09	*-*
	6	156,711	0.360	−0.825	1.11E + 05	1.07E-13	PRDM9*,* FANCA*,* SPIRE2*
	17	59,911,489 to 59,968,884	0.462	−1.294	1.07E + 05	1.30E-33	*SYCP2*
Crossover Interference (ν)	8	144,352	0.282	0.864	9.23E-02	8.15E-21	*RNF212*

*PRMD9 is annotated as *PRDM7*; see Discussion. ** >1 Mb away from top SNP.

## Discussion

This study has shown that individual variation in crossover count, positioning, and interference is heritable and differs between the sexes in a large breeding population of domestic pigs. A large number of sampled gametes allowed us to identify 14 distinct genomic regions associated with crossover trait variation in females. Crossover rate and interference were strongly negatively correlated and associated with a large effect locus corresponding to *RNF212*. Crossover positioning was partly correlated with crossover rate and was associated with large effect loci corresponding to *MEI4*, *PRDM9* and *SYCP2*. Crossover traits in males had lower heritabilities and no significant genomic regions; while this may be reflective of polygenic architectures, it may also be that the small number of unique males in this data set (271 males compared to 4,704 females) led to reduced power to identify moderate to large effect loci. Here, we discuss in more detail the role and function of candidate loci, variation in crossover interference, why genetic variation is maintained and potential breeding outcomes, and future directions for research.

### *RNF212* as a locus for crossover count and interference

Crossover count and interference showed strong associations with the locus *RNF212*. This locus and/or its paralogue *RNF212B*are consistently associated with crossover count in nearly every mammal study published to date^11,13,14,17–19^. Functional studies in mice have shown that RNF212 protein localises to recombination sites early in the crossover designation process, and is a dosage sensitive regulator of crossover formation^21^. The genetic correlation between crossover count and interference was strongly negative, and correcting interference for crossover count removed all association of *RNF212*. Therefore, another plausible mechanism is that lower crossover counts are mediated by higher crossover interference. This is suggestive of the coarsening model of crossover interference observed in *Arabidopsis thaliana*mediated by Hei10, a protein in the same family of E4 ligases which exhibit a conserved behaviour during meiosis^36,43^. In *A. thaliana*, a number of small Hei10 foci form on chromosomes during synapsis, which in turn aggregate into fewer, larger, distinct foci along the chromosome (i.e. they undergo a coarsening process), which correspond to final crossover sites^35,36^. Whilst we cannot directly infer the mechanism by which *RNF212* affects these processes in the pigs, our findings highlight a likely inter-dependence of crossover count and the strength of crossover interference in this population.

### Additional candidate loci associated with crossover count

Two additional genomic regions were associated with crossover count. One contained the locus *CTCF*, a DNA binding protein that is associated with the anchoring of chromatin loops and the boundaries of topologically associated domains^44^. There is evidence that CTCF contributes to the DNA loop and axis organisation of chromosomes during meiosis, and may interact with PRDM9 binding sites^45^. Another region contained the meiotic recombination proteins *REC8*, *CCNB1IP1* and *REC114*; this region has been association with crossover count in humans, deer and cattle^11,17,20^. *CCNB1IP1*is the orthologue of Hei10, and all three loci are required for the crossover process^46,47^, and there is evidence that *REC8*can be enriched at CTCF sites in mouse spermatocytes^48^.

### *SYCP2*, *MEI4* and *PRDM9* as loci for broad-scale crossover positioning

This study showed a strong association of the loci *MEI4* and *SYCP2*on crossover positioning, through intra-chromosomal shuffling and distance to the telomere. SYCP2 is an important component of the synaptonemal complex that is required for synapsis and recombination during meiosis, and may also play a role in the association between the centromere and the synaptonemal complex^49,50^. In mice, association of MEI4 to the chromosome axes is a requirement for meiotic DSB formation, and can be a limiting factor in this regard^51^, and there is evidence that MEI4 is directed to DSB sites by the PRDM9 protein^51^. MEI4 also forms a complex with REC114 (see above), which is essential for meiotic DSB formation^52^. *SYCP2* and *MEI4* were also shown to have a weaker but significant effect on crossover count, indicating that crossover positioning may also influence the number of crossovers that can occur. The association between *PRDM9*and broad-scale positioning is less clear, as this locus is generally considered as a mediator of the positioning of fine-scale recombination hotspots (i.e. in regions of 1–2kb)^53^. Different alleles of the *PRDM9*zinc-finger array bind to different sequence motifs throughout the genome; therefore, this locus could feasibly affect broad-scale positioning if there is a difference in motif abundances associated with broad-scale features of the genome^53^. In addition, the interaction of this locus with MEI4 may lead to broad-scale effects on top of its role in fine-scale variation. However, we did not find a significant interaction between genotypes of these two loci for intra-chromosomal shuffling or distance to the telomere (Table S5). Interestingly, crossover positioning also showed weak but significant effects with the loci *ZCWPW1* and *ZCWPW2*. *ZCWPW1* has co-evolved with *PRDM9*, and ZCWPW1 is recruited to recombination hotspots by PRDM9, and is required for DSB repair during meiosis^54^. In addition, in species where *PRDM9* has been lost, *ZCWPW1* and *ZCWPW2*are also more likely to be lost, suggesting a key role of both loci in the recombination process^55^. The region containing *ZCWPW1* also contained *STAG3*, a locus which is associated with all meiotic cohesion complexes and is required for normal axial localisation, DNA repair, and crossing over^56^.

### Variation and sex differences in crossover interference

Our dataset had large numbers of offspring for both male and female individuals, which allowed us to conduct an analysis of crossover interference within both sexes. We found that the strength of crossover interference was higher in male pigs, which also had the lower crossover rate; this pattern has also been found in humans, cattle, dogs and mice^37,57,58^. Crossover interference was modelled using a Houseworth-Stahl model of interference^40^, which allows both interfering and non-interfering crossovers to occur. Our results show that the number of crossovers escaping interference was not significantly different from zero. However, our crossover dataset may have reduced power to detect non-interfering crossovers, as (a) the median marker density for this analysis is around 1 SNP per 31.8 kb, and (b) the software used to estimate crossover positions (Lep-MAP3) uses a likelihood-based model to call crossovers based on phase changes, and may not call very short crossovers with marginal changes in map likelihoods^59^. Our analysis used sex specific genetic map distances rather than base pair distances to calculate crossover interference parameters. This choice was made as it is well-established that crossover interference operates on the length of the chromosome axis, i.e. physical length of the chromosome during pachytene^60^, and the synaptonemal complex length strongly correlates with genetic length in cM^24,61^.

#### Why is genetic variation for crossover processes maintained in pigs, and what are the implications for selective breeding?

Our findings add to a growing body of evidence of genetic variation underpinning crossover traits in domesticated mammal species^12,13,15,16,18,19^. Some models predict that strong selection (particularly in smaller breeding populations) should favour alleles that increase rates of recombination^62^, which should in turn reduce genetic variation in recombination rates. The heritability of the crossover traits measured is high in terms of FID mean values, but is low at the individual gamete level, most likely due to high within-individual sampling variance associated with (assumed) Mendelian segregation of daughter chromosomes with and without a given crossover. This means that there is evolutionary potential to modify crossover traits to reach an adaptive optimum. In addition, there is the capacity for independent evolution of crossover positioning and crossover rate, whereas interference and crossover rate are strongly genetically coupled in this population. Nevertheless, selection on crossover traits for their advantages related to breeding outcomes (i.e. through faster responses to selection) is likely to be weak. In species such as pigs, where there are a large number of chromosomes (2 *N* = 38), intra-chromosomal allelic shuffling contributes to a negligible amount of novel haplotypic variation at the genome-wide level when compared to the independent assortment of chromosomes (see Veller et al.^22^), . This is supported by a simulation study in hypothetical livestock breeding programme with 2 *N*= 20, where a two-fold increase in crossover count would only lead to breeding gains of 12.5% in polygenic traits^63,64^. The fitness consequences of increasing recombination rates to that level is not known, but there is compelling evidence that substantial increase in recombination is not beneficial. In humans, high levels of recombination have been associated with cancer^65^and mutation rates are known to be elevated in recombination hotspots^66,67^. Furthermore, it is not known whether such increases in number of crossovers are achievable due to other linked mechanisms in the meiosis machinery. It should also be noted that shifting from more terminal to more central crossovers is a more effective approach to increase allelic shuffling than increasing the overall crossover rate, but risks breaking up beneficial allelic combinations at a faster rate than selection could feasibly reach fixation. Overall, the utility of manipulating recombination for selective breeding is likely to be limited in domestic pigs; the mere presence of crossovers provides enough of a mechanism to purge deleterious mutations and to overcome Hill-Robertson interference in the face of genetic drift^62,68^.

## Conclusions

Our findings show that crossover rate, positioning, and interference can be driven by distinct genetic processes in domestic pigs. Future progress will rely on understanding the molecular mechanisms of this variation in more detail, particularly in terms of individual differences in chromosome architectures are the driver or consequence of genetic differences at the loci identified above. In addition, it remains to be known whether individual phenotypic and genetic variation in crossover traits is associated with individual fertility e.g. through proper segregation of chromosomes and maintenance of genomic integrity through reduced mutation rates associated with lower levels of DSB formation and repair.

## Methods

### Study population and genotype dataset

This study used genomic and pedigree data in a Large White breeding population of 95,613 individuals provided by Topigs Norsvin. All study individuals were genotyped on one of two SNP arrays: Illumina GeneSeek Custom 80 K SNP chip, and Illumina GeneSeek Custom 50 K SNP, with 50,705 SNPs in common between the two arrays (hereafter referred to as the 50 K dataset). Quality control removed SNPs with a minor allele frequency < 0.01, genotype call rate < 0.95, and strong deviation from Hardy Weinberg equilibrium ($\:{\chi\:}_{1}^{2}\:$> 600). The physical positions of the markers were determined relative to the Sscrofa11.1 reference genome assembly. The sex chromosomes were excluded from all analysis in this study. Key individuals in the pedigree were also genotyped on the Axiom porcine 660 K Affymetrix array, and genotypes for all focal individuals in this study were imputed to 660 K SNPs using the Fimpute genotype imputation software V2.2^69^. Quality control removed imputed SNPs with a minor allele frequency of < 0.01, with a final dataset of 524,587 SNPs. Note that the imputed dataset was only used in the genome wide association analysis and not to infer crossovers.

### Linkage mapping and Estimation of meiotic crossover positions

Individual crossover positions were estimated using the same approach as^15^using the 50 K dataset. Briefly, the pedigree was ordered into three generation full-sib families, consisting of two focal individuals (female and male), their parents, and their offspring. This allowed phasing of the 50 K marker dataset in focal IDs and their offspring, and consequently the identification of crossovers positions in gametes transmitted from the focal ID to their offspring. Characteristics of the crossover positions are then assigned as a phenotype of the focal ID, as this is where the meiosis took place. The software Lep-MAP3^59^ was used to construct sex-specific linkage maps at the population level, and to estimate autosomal crossover positions within individual phased gametes. Marker orders were assumed to be the same as Sscrofa11.1, and linkage maps were constructed in centiMorgans (cM) using the Morgan mapping function. Sample sizes are provided in Table 1.

### Estimation of crossover phenotypes

Four crossover phenotypes were estimated for each gamete for each focal ID (unless otherwise stated) using information on the meiotic crossover positions on autosomes. Each phenotype was determined in autosomes only to allow direct comparisons of rates between females and males. Differences between males and females within each phenotype were tested using Welch t-test^70^. Pearson’s correlation statistics were estimated between all crossover phenotypes within and across both sexes (Fig. 1). The phenotypes were as follows:

#### Crossover count

This measure is the total crossover count per gamete across all autosomes. Per gamete analysis of crossover count has previously been published for the same dataset in this breed^15^.

#### Intra-Chromosomal allelic shuffling (Crossover positioning)

Intra-chromosomal shuffling, $\:{\stackrel{-}{r}}_{intra}$, was calculated per gamete per focal ID as the probability that a pair of loci on the same chromosome are uncoupled due to a crossover event, using the following equation adapted from^22^.

$$\:{\stackrel{-}{r}}_{intra}=\sum\:_{k=1}^{n}2{p}_{k}(1-{p}_{k}){L}_{k}^{2}$$

where *k* is the autosome number 1–18, n is the number of autosomes, *p* is the proportion of paternally inherited alleles, *1-p* is the proportion of maternally inherited alleles, and *L* is the length of the chromosome as a fraction of the total length of the genome.

#### Distance from telomere

To obtain individual measures of crossover distance from telomere, we measured the physical distance between a crossover event and the closest telomeric end of the chromosome in Megabase pairs (Mb). On metacentric chromosomes, the distance from telomere for a crossover on the *p* arm was the position of the crossover in Mb, and distance from telomere for a crossover on the *q*arm was the total length of the chromosome minus the position of the crossover. If a chromosome arm had more than one crossover, only the crossover closest to the telomere was counted, i.e. a maximum 2 for metacentric chromosomes (1–12) and 1 for acrocentric chromosomes (13–18) and chromosomes with zero crossovers were excluded. For each gamete, we took the mean distance from telomere across chromosomes, resulting in multiple observations of distance from telomere for each focal ID. Centromere positions were obtained from^71^.

#### Crossover interference

Individual strength of crossover interference (n) was estimated following the Houseworth-Stahl interference escape model (also known as the gamma sprinkling or gamma escape model)^40^using the R package xoi v0.72 (Broman & Weber 2000). This model considers both Class I and Class II crossovers (where Class I crossovers are subject to interference, and Class II crossovers escape interference) and has been shown to be more robust than the gamma model, which considers only Class I crossovers^33^. For each focal ID, an estimate of n and the fraction of crossovers escaping interference (*p*) was obtained by fitting the *fitStahl* function on crossover positions in all gametes from that individual. This resulted in a single estimate of n and *p*for each focal ID. We used sex-specific cM positions rather than base pair (bp) positions, as interference acts on the length of the synaptonemal complex (SC) rather than sequence length. Recombination rates are highly correlated with SC length, and the relationship between SC length and base pair length varies between the sexes and along the genome in mammals^24^.

### Estimation of heritabilities and genetic correlations

Additive genetic variances were estimated for crossover phenotype within each sex separately using a restricted maximum-likelihood “animal model” approach^41^in ASReml-R v4^72^. For crossover rate and position traits, we modelled both (A) individual gamete phenotypes and (B) mean phenotype of all gametes of an FID. For crossover interference, only a single model was fit on the estimate per focal ID. The random effect structure included the additive genetic effect (fit with the inverse of the relationship matrix calculated from the pedigree). For models of individual gametes, we also modelled a permanent environment effect (the identity of the FID) as an additional random effect. The narrow sense heritability, h^2^_,_ was estimated as the fraction of the total phenotypic variance V_P_ that was explained by additive genetic variance V_A_. The additive genetic correlation, r_A_, was estimated using bivariate animal models between all crossover phenotypes within each sex (cross-phenotype correlations), and for each crossover phenotype between the sexes (cross-sex correlations).

### Genome wide association studies (GWAS)

The association between 524,587 imputed SNPs and each crossover phenotype within each sex was tested using the mixed linear model leaving-one-chromosome-out (MLM LOCO) model implemented in GCTA version 1.93.2 beta Linux^73^. This option performs an association analysis fitting a model where the chromosome on which the candidate SNP is located is left out when calculating the genomic relationship matrix. The model was fit as follows:

$$\:y=a+bx+g+e$$

where *y* is the crossover phenotype, *a* is the mean term, *b* is the additive fixed effect of the SNP currently tested for association, *x* is the genotype dosage (0,1,2) and *g* is the accumulated effect of all markers except the markers on the chromosome where the SNP currently tested for association is located. The genome-wide significance level was determined using a Bonferroni correction, with the threshold of α = 0.05 set at *P* = 9.53 × 10^−8^. Candidate loci in significant regions were determined using biomaRt v2.60.0^74^ in R v4.4.0 relative to Ensembl Release 112 to extract Gene Ontology terms for loci within 500 kb of the top associated SNPs. Gene names, GO names and GO descriptions were screened for the following text strings using the *grep* function: *meio*, *recombin*, *crossover*, *chromat*, *synapto*, *synapsis*, *gamet*, *double strand break*, *kinetoch*, *cohesin*, *histone*, *nucleosome*, and *spindle*. These flags were chosen to identify genes potentially associated with meiosis, synapsis, chromosome and chromatin structure, and gametogenesis. Selected candidates were screened for functions that matched the strings but not associated with meiosis (e.g. “synaptosome”). We then classified genes with GO terms matching the strings *meio*, *recombination*, *crossover*, *synaptonemal complex*, and *cohesin* as direct candidates, with the remainder classified as indirect candidates (Table S4).

For each crossover trait with two or more significantly associated regions, an interaction term was tested for between all pairs of loci associated with the trait. A linear regression model was fit with the crossover trait as the response variable and an interaction term between the genotype of the top SNP from each locus (0,1 or 2 of the effect allele) using the *lm* function from the stats package v4.2.3 in R v4.4.0.

## Electronic supplementary material

Below is the link to the electronic supplementary material.

Supplementary Material 1

Supplementary Material 2

Supplementary Material 3

## Data Availability

The raw data that support the findings of this study are available from Norsvin and Topigs Norsvin but restrictions apply to the availability of these data, which were used under license for the current study, and thus are not publicly available. However, data are available from the corresponding author upon reasonable request and with permission of Norsvin and Topigs Norsvin.
